# *In Vitro* Production of Echioidinin, 7-*O*-Methywogonin from Callus Cultures of *Andrographis lineata* and Their Cytotoxicity on Cancer Cells

**DOI:** 10.1371/journal.pone.0141154

**Published:** 2015-10-21

**Authors:** Arifullah Mohammed, Kishore K. Chiruvella, Yerra Koteswara Rao, Madamanchi Geethangili, Sathees C. Raghavan, Rama Gopal Ghanta

**Affiliations:** 1 Faculty of Agrobased Industry, Universiti Malaysia Kelantan, Jeli Campus, Locked bag-100, 17600, Jeli, Kelantan, Malaysia; 2 Department of Biochemistry, Indian Institute of Science, Bangalore 560 012, India; 3 Division of Plant Tissue Culture, Department of Botany, Sri Venkateswara University, Tirupati, Andhra Pradesh, India; 4 Department of Applied Chemistry, Chaoyang University of Technology, Taichung, Taiwan; University of New South Wales, AUSTRALIA

## Abstract

*Andrographis lineata* is an herbal medicinal plant used in traditional medicine as a substitute for *Andrographis paniculata*. Here, using mature leaf explants of *A*. *lineata* we demonstrate for the first time the callus induction established on MS medium containing 1.0 mg l^–1^ IAA. Dried callus was subjected to solvent extraction with acetone. Further the acetone residue was separated by silica gel column chromatography, crystallized and characterized on the basis of nuclear magnetic resonance (proton and c13) and liquid chromatographic mass spectroscopy. This analysis revealed the occurrence of two known flavones namely, 7-*O*-methylwogonin (MW) and Echioidinin (ED). Furthermore, these compounds were tested for their cytotoxicity against leukemic cell line, CEM. We identify that ED and MW induced cytotoxicity in a time- and concentration-dependent manner. Further increase in the LDH release upon treatment with ED and MW further confirmed our cytotoxicity results against leukemic cell line. Strikingly, MW was more potent than ED when compared by trypan blue and MTT assays. Our results recapitulate the utility of callus cultures for the production of plant specific bioactive secondary metabolites instead of using wild plants. Together, our *in vitro* studies provide new insights of *A*. *lineata* callus cultures serving as a source for cancer chemotherapeutic agents.

## Introduction

Among all types of cancer, leukemia is regarded one of the most aggressive life threatening hematological malignancies with millions of patients diagnosed each year. Leukemia is found to be very sensitive to chemotherapeutic agents [1]. Screening natural products derived from medicinal plants have been promising as valuable sources for antitumour drugs. Natural products exert anticancer potentials by inhibiting cell proliferation and inducing apoptosis [2,3]. Plant extracts which exhibit anticancer activity is mixture of all the compounds present in the extract instead of particular compound [4,5]. This urges for the search of isolated compounds with well-defined pharmacological properties. *In vitro* cultures employing plant tissue culture technology is advantageous over intact plant to produce secondary metabolites without destroying natural habitat [6,7,8]. This is due to the fact that the rate of cell growth and biosynthesis in culture initiated from a small amount of plant material is quite high, a considerably produced in a short period of time. Manipulating culture conditions, phytohormones is a valuable tool for increasing the level of bioactive metabolites [9]. This is in contrast to large number of *in vivo* plants used to obtain a small quantity of drug.

Production of plant-derived medicinal compounds is an issue of considerable socio-economic importance. This has prompted industries, as well as scientists to consider the usage of *in vitro* cultures as an alternative supply. Production of plant secondary products by growing undifferentiated tissues *in vitro* large amounts have long been recognized as a means in which both seasonality and time specificity of production would be circumvented. Callus cultures are promising to obtain plant-specific valuable metabolites exploited to enhance the yield of bioactive compounds, to expedite the pace of conservation and propagation of an important ethno-medicinal plants [10]. Previous studies have succeeded in the production of secondary metabolites through *in vitro* culture [11,12,13,14,15,16]. For example, prenylated flavanones such as sophoraflavanone G and lehmanin were obtained from *Sophora flavescens* callus culture [17]. Callus of *Hypericum perforatum* yeilded 6-C-prenyl luteolin, along with luteolin-5,3’-dimethyl ether, luteolin-5-glucoside and luteolin-3’- glucoside [18]. Importantly anticancer compound such as taxol was also obtained from callus cultures of Taxus species [19,20,21]. Strikingly, isoflavone content from callus cultures of Genista species were more than the *in vivo* plants [22,23].

*Andrographis* (Acanthaceae) comprises of 250 genera and 2500 species. Among all the genera, Andrographis *paniculata* is of potential significance in traditional medicine for treating various diseases due to its wide spectrum of biological activities [24,25]. The pharmacological activities of *Andrographis* are due to the presence of flavonoids, terpenoids and flavonoid glycosides like andrographolide, echiodinin and echiodinin 5-*o*-β-D-glucopyranoside. Andrographolide and their derivatives possess potential anticancer properties [26]. 7–*O*–methyl dihydrowogonin, 5–hydroxy–3,7,8,2′–tetramethoxy flavone and flavone 7–*O*–methylwogonin from the roots of *A*. *paniculata* have been identified [27]. Callus cultures of *A*. *paniculata* were reported to be 7–*O*–methylwogonin, skullcap flavone I and 5–hydroxyl–7,8,2′–trimethoxy flavone [28]. The occurrence of 7–*O*–methylwogonin constitutes the first report of a 2′–deoxyflavone.

*Andrographis lineata* used in the present study is a tribal medicinal plant serving as good substitute for *A*. *paniculata*. Similarly this plant is used by medical practitioners for treating snake bites, diabetes, skin diseases, fever, constipation, bronchitis, cancer, inflammation and stomachic. It also possesses antihelminthic, antiinflammatory, antipyretic and antiperiodic properties [29,30]. Leaf paste is used for curing respiratory infections while root decoction as antidote. Flavonoids along with andrographolide have been reported from leaf extracts [31]. Pharmacological studies with leaf extracts displayed antibacterial, diuretic hepatoprotective and antidiabetic activities [32,33,34].

Previous studies with *A*. *paniculata* have demonstrated the utility of callus cultures for the production of secondary metabolites instead of using wild plants. This prompted us to develop callus induction for *in vitro* production of secondary metabolites in a relatively short period of time by passing seasonal pressure round the year. Here we report for the first time establishment of callus cultures from leaf explants of *A*. *lineata*. We show isolation and structural characterization of echioidinin (ED) and 7-*O*-methywogonin (MW) by silica gel column chromatography followed by nuclear magnetic resonance (proton and c13) and liquid chromatographic mass spectroscopy. Further we demonstrate ED and MW induced cytotoxicity against leukaemic cells in a time- and concentration-dependent manner.

## Materials and Methods

### Reagents and Chemicals

Chemicals such as NAA, 2,4-D, IAA were obtained from Sigma. HgCl_2_ was from E.Merck, sucrose from Sisco, silica gel from Acme synthetic chemicals and Agar from CDH, Mumbai, India. DMSO, DMF, acetone, hexane, ethyl acetate and methanol were obtained from Sigma.

### Plant Material and Tissue Culture Conditions for Callus Induction

Mature leaves collected from field grown plants of *A*. *lineata* were used as explants. The leaves were surface sterilized in 70% ethanol for 30 sec followed by rinsing in sterile distilled water, then treated with 0.1% HgCl_2_ (w/v) (Merck) for 2 min and followed by washing in sterile distilled water. The cut ends of the explants were trimmed with sharp edge sterile surgical blades. Further, the explants were blotted on sterile filter paper discs before inoculation. Nutrient media used in the present study was Murashige and Skoog's medium, 1962. The media were congealed with agar (0.8%), and sucrose 3% was used as a source of carbohydrate. The pH of the medium was then adjusted to 5.6–5.8 and the medium was finally made to a known volume. Agar was added to the media before dispensing into the containers (15 ml for 25 × 150 mm test tubes) which were autoclaved for 15 min at 15 lbs/in^2^. The test tubes containing sterile media were placed in a slanting position in order to increase the surface area of the media. All cultures were incubated in a culture room at 25 ± 2°C with a relative humidity of 50–60% and 16 h photoperiod at a photon flux density of 15–20 μ E m^2^ s^–1^ from white cool fluorescent tubes.

MS medium with different concentrations of auxins (2,4-D, IAA and NAA) ranging from 0.1–1.0 mgl^- l^ were used to study their effects on callus induction at varying concentrations (Table 1). Subsequently, the well-established callus obtained on MS medium fortified with 1.0 mg l^–1^ IAA was subcultured 4–5 times for optimum callus production at regular intervals of 20 days after inoculation. This technique of callus induction for production of secondary metabolites has been previously described by various groups [10,14,23,35].

**Table 1:** Effect of auxins on callus induction from leaf explants of *Andrographis lineata* after 4 weeks of culture.

2,4-D	IAA	NAA	Callus	
(mg l^–1^)			frequency (%)	Nature
0.1	-	-	41.2	White creamy soft friable callus
0.3	-	-	53.4	White creamy soft friable callus
0.5	-	-	61.3	White creamy soft friable callus
1.0	-	-	36.5	NR
-	0.1	-	36.2	White creamy soft friable callus
-	0.3	-	48.2	White creamy soft friable callus
-	0.5	-	59.4	White creamy soft friable callus
-	1.0	-	75.0	Light green hard compact nodular callus
-	-	0.1	31.3	Yellowish hard compact nodular callus
-	-	0.3	35.1	Creamy yellowish soft friable callus
-	-	0.5	43.2	Yellowish soft friable callus
-	-	1.0	49.3	NR

NR indicates no response. Data represents 20 replicates and the values recorded after two weeks of culture initiation

### Extraction, Isolation and Identification of Purified Compounds from *A*. *lineata* Calli

**Fig 1:**
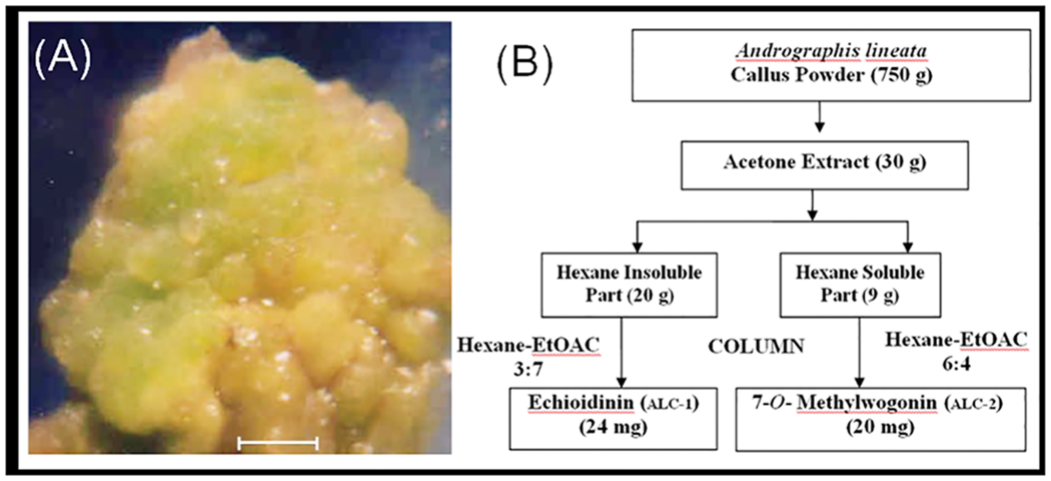
Callus induction from mature leaf explants of *Andrographis lineata* on MS medium. (A) Callus was induced from leaf explants on MS medium supplemented with 1.0 mg l^-1^ IAA (Bar = 2.5 mm) after 4 weeks. (B) Schematic representation of phytochemical analysis of callus of *Andrographis lineata* for isolation of bioactive compounds.

The procured calli was first dried at room temperature for a few days and the material was then crushed to a fine powder (750 g). The powder obtained was subjected to solvent extraction using hot acetone in a soxhlet appartus [10]. Hot extraction enhances the solute solubility of natural products and, extracts most of the secondary metabolites. In order to obtain the maximal number of compounds from *Andrographis lineata* calli, we used acetone for soxhlation under hot condition. Additionally, usage of acetone as extracting solvent is not to extract lipids with the same efficiency as it would for secondary metabolites. Evaporation of the extract *in vacuo* yielded a dark brown residue, acetone extract (30 g) (Fig 1B). The acetone extract suspended in H_2_O, was partitioned with hexane to give hexane soluble and insoluble fractions. Further, we carried out hexane fractionation as it removes out chlorophyll and non-polar constituents.

The hexane insoluble residue (20 g) was chromatographed over silica gel using hexane–ethyl acetate gradient eluent, and similar fractions were combined to produce four sub-fractions (Fr. I to Fr. IV). Fractions II was further separated using a silica gel column eluting with a gradient of hexane−ethyl acetate to afford a yellow solid (24 mg) which was designated as ALC–1. This gave a green color with Fe^3+^ an orange red color with both Mg–HCI and Zn–HCI. On the other hand, repeated silica gel CC (5×90 cm) of the hexane soluble fraction with increasing polarity using mixtures of ethyl acetate/hexane gave three sub-fractions (F1–F3). Fraction F2 was rechromatographed over a silica gel column using hexane/EtOAc (60:40) to afford a white solid (20 mg). Further this on crystallization from methanol afforded colourless needles (19 mg). This was designated as ALC–2. It gave a green color with alcoholic ferric chloride and a pink color with Mg–HCl acid.

Further purification of subfractions from hexane insoluble (Fr. I, Fr. III–IV), and hexane soluble (F1 and F3) fractions did not yield any crystalline principle. All the eluates that contained “residue” or “no residue” were examined by TLC as described below by spraying 8% methanolic sulfuric acid. This is followed by heating on a hot plate at 100°C for 4 min for optimal color development. The plates were visualized and Rf values were calculated. Next the purified compounds were eluted into the corresponding solvent systems and the eluates were preserved for spectral analysis.

Ultra violet (UV) spectra were recorded on Beckman 25 and Shimadzu UV–240 spectrophotometers and values were given in nm. Infra-red (IR) spectra were recorded on Perkin–Elmer model 283B and 297 double beam spectrophotometers and *ν* values were given in cm^−1^. Proton magnetic resonance (^1^H NMR) spectra were recorded on Varian, 200, JEOL FX–90Q and Bruker–AM–300 spectrophotometers with TMS as internal standard. Carbon–13 nuclear magnetic resonance (^13^C NMR) spectra were recorded on JEOL FX–90Q spectrophotometer. The chemical shifts were given in δ ppm. Mass spectra (MS) were recorded on LC–MSD–Trap–SL (Agilent Technologies) at National Center for Mass Spectroscopy at Indian Institute of Chemical Technology, Hyderabad.

### Thin Layer Chromatography (TLC)

The glass plates of (20 × 20 cm) were washed thoroughly under running tap water followed by distilled water and the plates were kept ready for silica gel application. Silica gel 25 g (acme) was dissolved in 50 ml of double distilled water, stirred well and the slurry was then passed in the spreader. The spreader was gently pulled along the plates, (2–3 mm thickness) to get an even application. The plates were activated at 100°C for about 45 min, allowed to cool, removed from the oven and subjected to use.

The eluates were applied in the form of spot with micropipette, 2 cm above the base of the plate and were allowed to dry with hair dryer. Then the plates were developed in hexane:ethyl acetate 9:1 (v/v) and in hexane:ethyl acetate 8:2 (v/v). The solvent was allowed to move up to 15 cm, then were removed, allowed to dry. The plates were sprayed with 8% methanol sulfuric acid, there after subjected to heating in a hot air oven at 100°C for 5 min. Then the compounds were visualized and Rf values were calculated. Similarly prior made silica gel coated plates was developed in different solvent systems with hexane, ethyl acetate and methanol extracts. The plates were opposed to the chromatoplates received the spray reagent. The outline of the compounds was marked with a needle on the unsprayed silica gel plates.

### Cell Culture

Human leukemia cell line CEM was purchased from National Center for Cell Science, Pune, India. Cells were grown in RPMI 1640 (SERA LAB, USA) containing 10% FBS (GIBCO BRL, USA), 100 U of Penicillin G/ml and 100 μg of streptomycin/ml at 37°C in a humidified atmosphere containing 5% CO_2_. Cells were split at the ratio of 1:10 every 3–5 days.

Echioidinin (ED) and 7-*O*-methywogonin (MW) used in the present study are natural flavonoids purified from leaf callus of *A*. *lineata*. Compounds ED and MW was dissolved in DMF (Sigma, USA). The maximum concentration of DMF (Dimethylformamide) used in the experiments was equal to 0.05% and the same amount was used as vehicle control. In all the experiments described herein echioidinin and 7-O-methywogonin was added 24 h after the culture.

### Cell Viability by Trypan Blue Exclusion

Trypan blue exclusion assay was performed to assess the effect of ED and MW on viability of CEM cells. Approximately 0.75×10^5^ cells/ml was treated with different concentrations of ED and MW. Following 24 h of cell growth, different concentrations of ED and MW (10, 50, 100, or 250 μM) or vehicle (DMF) were added to the cells. Cells were withdrawn from the culture after every 24h and stained with trypan blue as described [2,36]. The number of cells (viable-unstained and non-viable-blue) were counted using hemocytometer. Each experiment was done three independent times with good agreement.

### Cell Proliferation by MTT assay

Cell survival was further assessed by 3-(4,5-dimethylthiazol-2-yl)-2,5-diphenyl tetrazolium bromide (MTT) dye reduction assay, which is based on the ability of viable cells to metabolize a yellow tetrazolium salt to violet formazan product that can be detected spectrophotometrically. CEM cells growing at log phase were treated with different concentrations of ED and MW (10, 50, 100, 250 μM) and incubated in 5% CO_2_ atmosphere with high humidity. Cells were collected after 48 and 72h and treated with MTT (0.5 mg/ml) as described earlier [36,37]. Absorbance was measured at 570 nm on a multiwell ELISA plate reader. The mean absorbance of medium controls was the blank and was subtracted. Concentration of compound causing 50% inhibition of cell growth (IC_50_) was estimated after 72h of exposure. The absorbance of control cells were taken as 100% viability and the values of treated cells were calculated as a percentage of control. Data shown is obtained from three independent batches of experiments.

### LDH Release assay

Release of lactate dehydrogenase (LDH) is an indicator of membrane integrity and hence, cell injury. LDH assay was performed to assess the LDH release in the culture following ED and MW treatment (10, 50, 100 and 250 μM) on CEM cells for 48 and 72h as per standard protocols [37]. The intracellular LDH was determined after lysing the cells by freezing and rapid thawing. The LDH release was measured at an absorbance of 490 nm. The percentage of LDH release was calculated as: (LDH activity in media)/(LDH activity in media + intracellular LDH activity)×100. Each experiment was done three independent times with good agreement.

### Statistical Analysis

The results are expressed as the mean plus or minus standard error. All analyses were performed with the GraphPad software using one-way ANOVA followed by Tukey-Kramer Multiple Comparison Test. Statistical significance was considered at p<0.05.

## Results

### *In vitro* Callus Induction From Mature Leaf Explants

We established callus cultures from mature leaf explants on MS medium fortified with auxins like NAA, 2,4-D and IAA. The nature of the callus varied with the concentration of different auxins used (Table 1). White, soft and friable callus was obtained on MS medium supplemented with 2,4–D (0.1–0.5 mg l^–1^). The frequency of light yellowish green, hard, organogenic callusing in terms of biomass growth was found to be maximum (75%) in presence of 1.0 mg l^–1^ IAA (Fig 1A) after 4 weeks (Table 1).

However, 1.0 mg l^–1^ NAA and 2,4-D greatly exacerbated the frequency of callus formation. The age of the leaf explant was critical for callus continued proliferation. Usage of older leaves lowered callus formation. The optimum callus induction was observed in presence of 1.0 mg l^–1^ IAA and was used for the isolation of bioactive compounds (Fig 1A). Organogenic callus was increased in its volume by subculturing callus segments on MS medium fortified with 1.0 mg l^–1^ IAA for every twenty days. Callus was healthy during all subcultures and subculturing of callus provided bulk quantity for secondary metabolite extraction. As expected, upon transfer to media containing cytokinin, BA showed shoot morphogenic response (data not shown).

### Structure Identification of Echioidinin and 7–*O*–methylwogonin

Characterization and structural determination of echioidinin (ED) and 7-*O*-methywogonin (MW) were established mainly on the basis of 1D NMR and mass spectral studies. The compound ALC–1 was crystallized from methanol as yellow crystals (20 mg) with the melting point, 264–265°C. It was analysed for its molecular formula of C_16_H_12_O_5_ with molecular weight of 285 [M+H]^+^. It was purple under UV and UV/NH_3_. The spectral details of the compound are given below.

**Fig 2:**
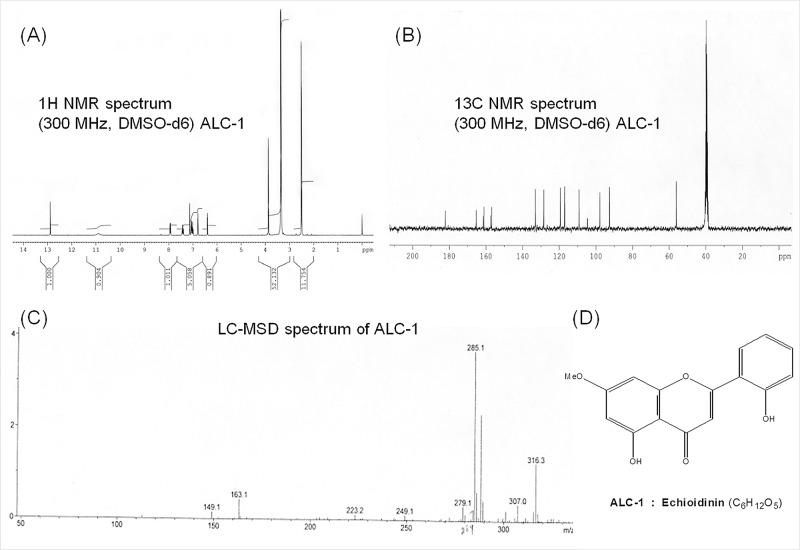
Structural elucidation of echioidinin (C_16_H_12_O_5_) by spectral analysis. (A) ^1^H NMR spectra (B) ^13^C NMR spectra (C) Liquid chromatography mass spectra (D) Structure of echioidinin.

UV (S1A Fig): *λ*_max_ (MeOH) (log ε) 265(4.40), 335 (4.19) nm. IR (S1B Fig): *v*_max_ (KBr) 3416 (–OH), 2980, 1662 (>C = O), 1614, 1504, 1452, 1350, 1245, 1159, 865, 831 cm^−1^. ^1^H NMR (Fig 2A): (300 MHz, DMSO–*d*_6_) δ 12.88 (1H, s, 5–OH, exchangeable with D_2_O), 10.90 (1H, s, 2′–OH, exchangeable with D_2_O), 7.90 (1H, dd, *J* = 2.0, 8.0 Hz, H–6′), 7.40 (dt, 1H, *J* = 2.0, 8.0 Hz, H–4′), 7.10 (s, 1H, H–3), 6.99–7.05 (m, 2H, H–3′, 5′), 6.75 (d, 1H, *J* = 2.5 Hz, H–8), 6.35 (d, 1H, *J* = 2.5 Hz, H–6), 3.90 (s, 3H, OMe–7). ^13^C NMR (Fig 2B): (300 MHz, DMSO–*d*_6_) δ 182.0 (C–4), 165.1 (C–7), 161.4 (C–2), 161.0 (C–5), 157.3 (C–8a), 156.7 (C–2′), 132.8 (C–4′), 128.4 (C–6′) 119.3 (C–5′), 117.0 (C–1′), 116.9 (C–3′), 109.1 (C–3), 104.6 (C–3a), 97.8 (C–6), 92.4 (C–8), 55.9 (OMe–7). LC–MSD (Fig 2C): *m*/*z* = 284 [M]^+^, 285 [M+H]^+^.

Thus, from the above foregoing spectral studies ALC–1 was characterized as 5, 2′–dihydroxy–7–methoxyflavone (Echioidinin, ED) (Fig 2D) as its physical and spectral data was in good agreement with literature values [38].

Next, compound ALC–2 was crystallized from hexane as pale yellow needles (20 mg) with melting point, 181–182°C. It was analysed for its molecular formula of C_17_H_14_O_5_ with molecular weight, 298. It was purple under UV and UV/NH_3_. The spectral details of the compound are given below.

**Fig 3:**
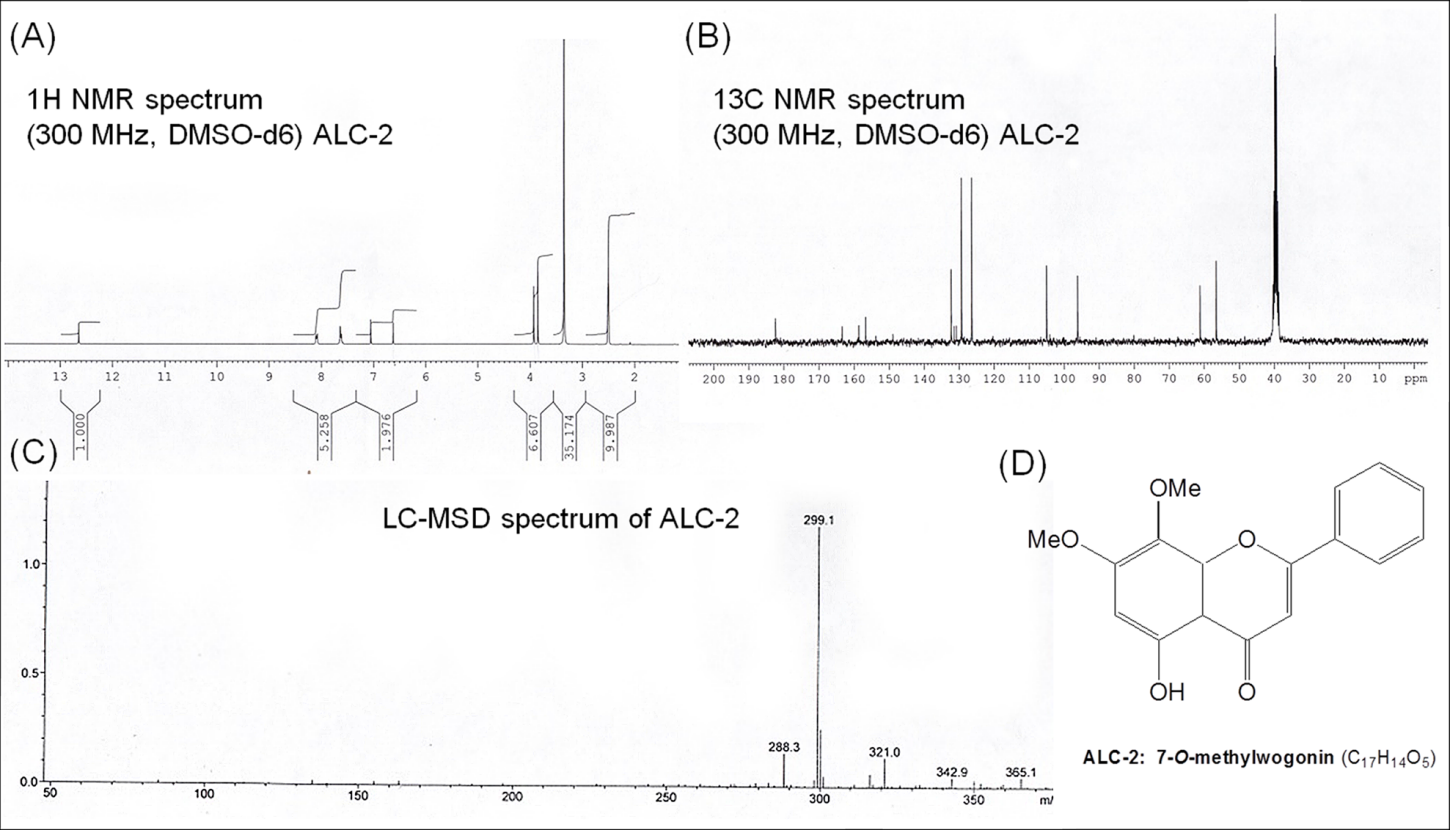
Structural elucidation of 7–*O*–methylwogonin (C_17_H_14_O_5_) by spectral analysis. (A) ^1^H NMR spectra (B) ^13^C NMR spectra (C) Liquid chromatography mass spectra (D) Structure of 7-*O*-methyl wogonin.

UV (S2A Fig): *λ*_max_ (MeOH) nm (log*ε*) 272 (4.52), 345 (3.78) nm. IR (S2B Fig): *ν*_max_ (KBr) 3416 (–OH), 2938 (–OMe), 1661 (>C = 0), 1605, 1510, 1447, 1376, 1274, 1225, 1125, 1030, 970, 833, 767, 686 cm^−1^. ^1^H NMR (Fig 3A): (300 MHz, DMSO–*d*_6_) δ 12.65 (1H, s, OH–5), 8.10 (2H, m, H–2′, 6′), 7.61 (3H, m, H–3′, 4′, 5′), 7.01 (1H, s, H–3), 6.60 (1H, s, H–6), 3.98 (3H, s, OMe–7), 3.87 (3H, s, OMe–8). ^13^C NMR (Fig 3B): (300 MHz, DMSO–*d*_6_) δ 183.5 (C–4), 164.7 (C–2), 160.0 (C–7), 159.4 (C–5), 150.3 (C–8a), 132.8 (C–4′), 132.3 (C–8), 130.0 (C–1′), 130.1 (C–3′, 5′), 127.2 (C–2′, 6′), 105.8 (C–3), 105.4 (C–4a), 96.7 (C–6), 61.6 (OMe–8), 56.8 (OMe–7). LC–MSD (Fig 3C): *m*/*z* = 299.1 [M+H]^+^.

Thus from the foregoing spectral studies, the structure of ALC–2 was elucidated as 5–hydroxy–7, 8–dimethoxyflavone (7–*O*–methylwogonin) (Fig 3D) and was also confirmed by comparison of literature values [39].

### Echioidinin (ED) and 7–*O*–Methylwogonin (MW) Induced Cytotoxicity in Leukemic Cells in a Dose- and Time-Dependent Manner

**Fig 4:**
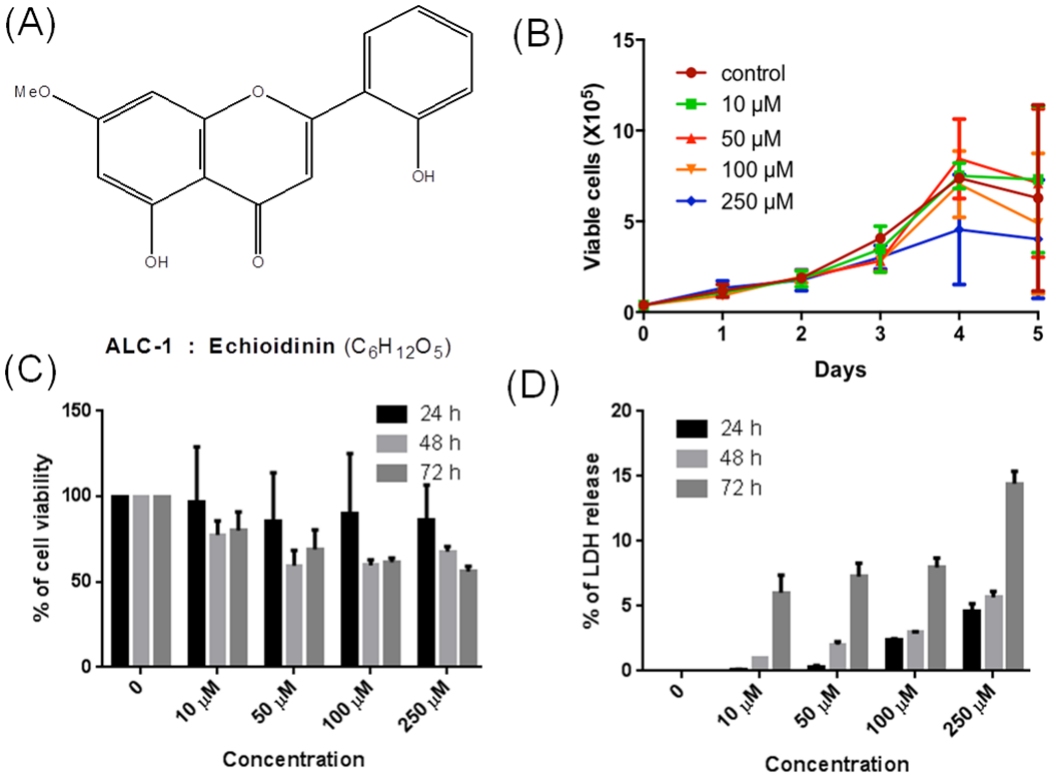
Cytotoxicity analysis of echioidinin (ED) on leukemic cell line, CEM. (A) Structure of ED. (B) Evaluation of cell viability using trypan blue assay following 10, 50, 100 and 250 μM of ED or DMF (control) treatment. Cells were harvested, trypan blue stained and counted every 24 h until it reached stationary phase. (C) Determination of % cell viability by MTT assay in CEM cells. Cells were cultured with 10, 50, 100 and 250 μM of ED or vehicle control for 24, 48 and 72 h. The % of cell viability was calculated considering DMF treated cells as 100% and plotted with representation of error bars. (D) Measurement of LDH release following treatment with ED. After the exposure of CEM cells with ED at different concentrations (10, 50, 100 and 250 μM) for 24, 48 and 72 h, the release of LDH was measured at 490 nm. Results are presented as percentage of LDH release. Each experiment was done three independent times with good agreement.

**Fig 5:**
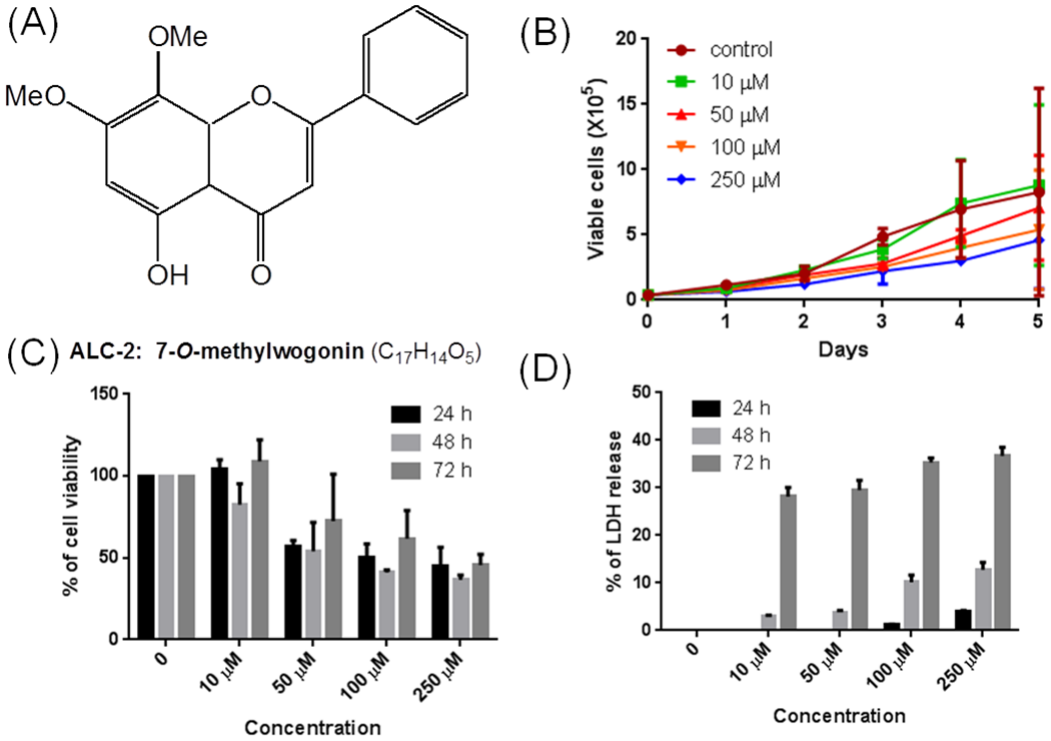
Cytotoxicity analysis of 7-*O*-methylwogonin (MW) on leukemic cell line, CEM. (A) Structure of MW. (B) Evaluation of cell viability using trypan blue assay following 10, 50, 100 and 250 μM of MW or DMF (vehicle control) treatment. Cells were harvested, trypan blue stained and counted every 24 h until it reached stationary phase. (C) Determination of % cell viability by MTT assay in CEM cells. Cells were cultured with 10, 50, 100 and 250 μM of MWor vehicle control for 24, 48 and 72 h. The % of cell viability was calculated considering DMF treated cells as 100% and plotted with representation of error bars. (D) Measurement of LDH release following treatment with MW. After the exposure of CEM cells with MW at different concentrations (10, 50, 100 and 250 μM) for 24, 48 and 72 h, the release of LDH was measured at 490 nm. Results are presented as percentage of LDH release. Each experiment was done three independent times with good agreement.

Next, we examined the cytotoxic effect of ED and MW (Figs 4A and 5A) on the proliferation and survival of leukemic cell line, CEM (T-cell leukemia). Trypan blue assay was the first line of our investigation, where CEM was treated with 10, 50, 100 or 250 μM of ED and MW. The cells without addition of compound served as control. Since the compound was dissolved in DMF (Dimethylformamide), the cells with DMF were used as vehicle control. The maximum concentration of DMF used in the experiments was equal to 0.05% and the same amount was used as vehicle control. Following addition of compound, cells were counted at intervals of 24 h until the control cells attained stationary phase. Results showed that cell growth was affected with increase in time (Figs 4B and 5B). The effect was limited when 10 μM ED and MW was used. However, concentrations of 100 and 250 μM resulted in increased cell death (Figs 4B and 5B). The IC_50_ of ED and MW was approximately 100 μM, at 72 h of treatment.

The cytotoxicity induced by ED and MW on proliferation of leukemic cells was further verified using MTT assay. CEM cells treated with 10, 50, 100 and 250 μM of ED and MW were harvested after 24, 48 or 72 h and were subjected to MTT assay (Figs 4C and 5C). Results showed that cell viability was affected upon treatment with ED and MW at 100 and 250 μM, especially after 72 h. These results further suggest that ED and MW induce the cytotoxicity.

Further, LDH assay was carried out to determine the cellular integrity following ED and MW treatment (10, 50, 100 and 250 μM). Results showed a concentration- and time-dependent increase in the LDH release upon treatment with ED and MW, further confirming the above results (Figs 4D and 5D). Overall, our results suggest that ED and MW were capable of inducing cytotoxicity in cancer cell line.

## Discussion

There has been a growing interest to identify plant products as therapeutic agents that can effectively interfere with cancer cell proliferation. One striking medicinal plant in this register is *A*. *lineata* with a wide range of pharmacological effects. In the present study firstly, we describe establishment of callus cultures from mature leaf explants. Secondly, we demonstrate isolation and structural characterization of echioidinin, 7-*O*-methywogonin from callus cultures. Third, utilizing cell based assays we display treatment of echiodinin, 7-*O*-methywogonin on leukemia cells abolished its cell proliferation. The above conclusions drawn are based on several different analyses as described in our paper. These results are important due to realizations for humans as the search for new pharmacologically active compounds from plants has led to the discovery of many clinically useful drugs.

Several lines of evidence in the past suggests the role plant growth regulators stimulating the biosynthesis of medicinally important secondary metabolites in plant *in vitro* tissue cultures [10,22,35,40]. Such secondary metabolite production *in vitro* is based on the premise that the valuable product produced in nature in any organ or other tissue from a plant can be stimulated to accumulate in undifferentiated cells. Such callus cultures are capable of producing metabolites in quantities well above the limit of detection, depending on nutrient medium, sucrose concentration, age of the callus and cell line. Wewetzer [40] reported that morphological differentiation is not a prerequisite for azadirachtin production; however the highest concentrations were detected in completely undifferentiated cells. We report for the first time IAA accumulating the flavonoids in callus culture of *A*.*lineata*. Recently, it has been reported the influence of IAA in increasing the isoflavone content in callus cultures of *Genista tinctoria* along with cytokinin [22]. Further our observation of green spots on callus indicate the high callus growth rate as well callus ability to produce shoots. Researchers have succeeded in producing several valuable secondary phytochemicals in unorganized callus cultures [14,35,41,42,43,44,45].

In the present study, a systematic phytochemical investigation of *A*. *lineata* callus cultures lead to the isolation and characterization of two compounds: 5, 2′–dihydroxy–7–methoxyflavone (echioidinin) and 7–*O*–methylwogonin. Isolation of 2′–oxygenated flavonoids, which occur rarely in nature, from *A*. *lineata* provided strong evidence that “*Andrographis* species are noted for profuse production of 2′–oxygenated flavonoids and andrographolide diterpenoids” [46]. Compounds isolated from Acanthaceae so far were confined to *Andrographis* species only; this shows promise of being a useful chemotaxonomic marker for *Andrographis* in the Acanthaceae. The extraction of callus in different solvents in the present study as carried, in order to extract out all non-polar and polar constituents. The compounds were characterized using spectroscopic techniques like IR, 1D NMR and LC-MS. The above analysis shows that improved *in vitro* plant tissue culture systems have potential for commercial exploitation of secondary metabolites. However, production and extraction have to be increased many fold before large-scale production can be considered seriously.

Leukemic cells lines serve as useful tools to study factors and processes associated with their differentiation and apoptosis pathways [1]. Hence, cytotoxic effects of ED and MW on CEM cells (leukemic cell line derived from T-cell leukemia patient) is studied. Our results suggest that ED and MW induced cytotoxicity in human leukemic cells in a dose- and time-dependent manner. This study is useful as the search for natural products has been tremendously useful as cancer chemotherapeutic agents. The isolated compounds in this study ED and MW were plant derived constituents and their observed cytotoxicity in leukemic cells was of importance. Induction of cell death is important property of chemotherapeutic agent.

Cytoxicity of cancer cell death occurs by apoptosis and necrosis. Trypan blue assay is used for staining dead cells and cannot distinguish between healthy cells and cells that are alive that have lost cell function. Hence MTT assay was performed for determining mitochondrial dehydrogenase activity in living cells. LDH assay reflects cell condition with more sensitivity as this assay depends on several factors such as hydrogenase, NAD(H), NADP(H) and mitochondrial activity. MTT assay is cannot differentiate cell cycle/cell growth inhibition and cellular death. LDH can demonstrate the percentage growth inhibition as well as the percentage of killing. A hall mark for necrotic cells is the permeabilization of plasma membrane which in present study was quantified by the release of the lactate dehydrogenase (LDH), which is the leakage of intracellular molecules through impaired plasma membrane. LDH is a soluble cytoplasmic enzyme that is present in almost all cells and is released into extracellular space when the plasma membrane is damaged. However LDH release assay cannot distinguish between primary necrosis and secondary necrosis as a consequence of apoptotic cell death. Thus for the possible treatment of cancer, cytotoxic compounds should preferentially act via apoptosis.

Pharmacologically, MW is known to Inhibit activity of adenosine 3,5-cyclic monophosphate phosphodiesterase [47] while ED effects on respiratory system, decreases the incidence of common colds and relieves from pharyngotonsilltis [48]. Although ED and MW induced cytotoxicity and necrosis in CEM cells, the IC_50_ value is higher than many synthetic compounds. In fact, previous reports showed that similar doses were also used for other compounds [49,50]. Despite the obvious limitation, the data of this study can be useful for the development of chemotherapeutic agents.

In conclusion, Echioidinin and 7-*O*-methywogonin produced *in vitro* suggests the utility of callus cultures for production of bioactive secondary metabolites instead of using wild plants. Further our study demonstrates a promising alternative strategy for the conservation natural plant resources, importantly to overcome the limited availability. Besides, our study provides new insights of callus cultures of *A*.*lineata* inducing cytotoxicity on cancer cells. However, more studies are essential to unravel their mechanism and importantly further studies are required to derive molecules with lower IC_50_ values that can be used as potential anticancer drugs.

## Supporting Information

S1 Fig Structural elucidation of Echioidinin (C_16_H_12_O_5_) by spectral analysis. (A) UV spectra (B) IR spectra.
(TIF)

S2 Fig Structural elucidation of 7–O–Methylwogonin (C_17_H_14_O_5_) by spectral analysis. (A) UV spectra (B) IR spectra.
(TIF)

## Abbreviations

MS: Murashige and Skoog
BA: benzyladenine
IAA: indole 3 acetic acid
NAA: naphthalene acetic acid
2,4-D: 2,4-dichlorophenoxyacetic acid
TLC: thin layer chromatography
NMR: nuclear magnetic resonance
LC/MS: liquid chromatography/Mass spectrometry
UV: ultraviolet visible spectroscopy
IR: infrared spectroscopy
MTT: 3-(4,5-dimethylthiazol-2-yl)-2,5-diphenyltetrazolium bromide
MW: 7-*O*-methylwogonin
ED: echoidinin
DMF: dimethylformamide
DMSO: dimethylsulphoxide
nm: nanometer
MHz: mega hertz
