# Supplementary figures and images for: In Vitro Production of Echioidinin, 7-O-Methywogonin from Callus Cultures of Andrographis lineata and Their Cytotoxicity on Cancer Cells
Source: PLoS One. 2015 Oct 21;10(10):e0141154. doi: 10.1371/journal.pone.0141154 (PMC4619555; doi:10.1371/journal.pone.0141154)

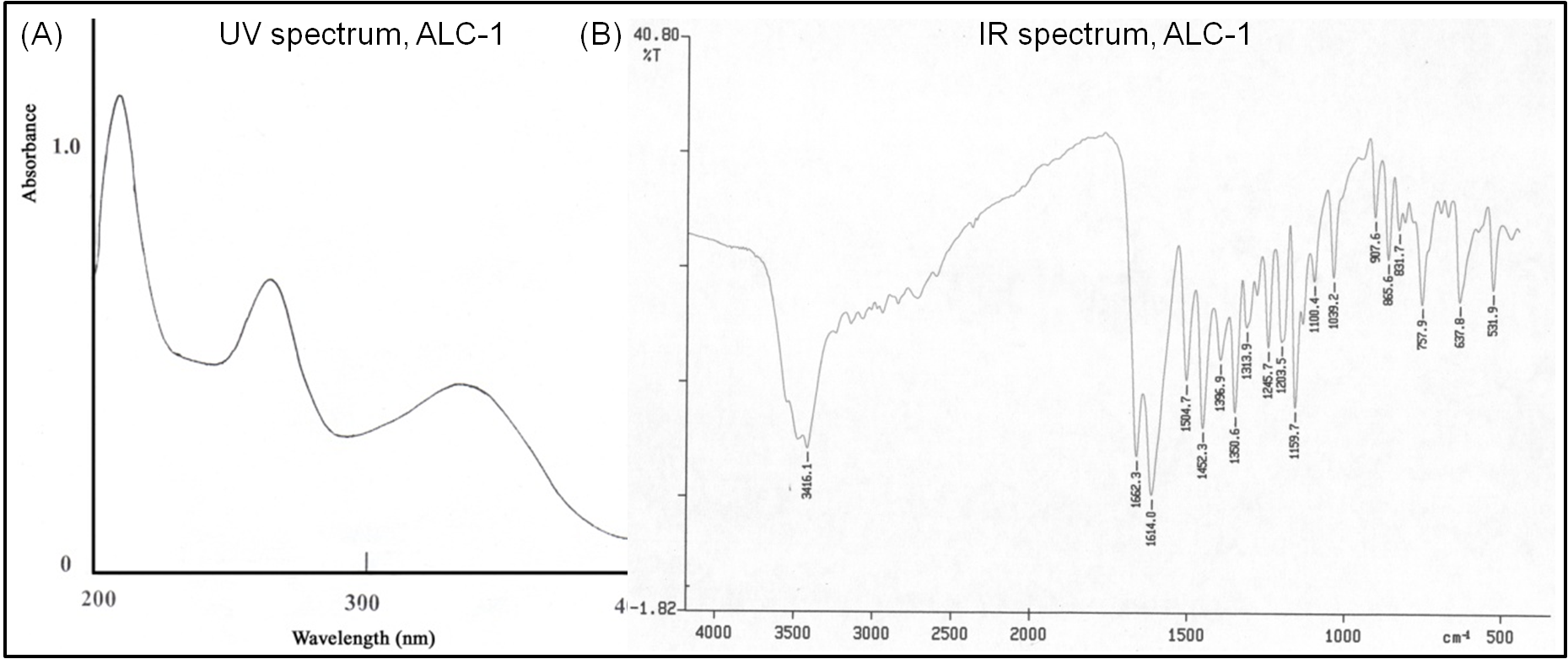

Supplement: S1 Fig — (A) UV spectra (B) IR spectra. (TIF) [file pone.0141154.s001.tif]

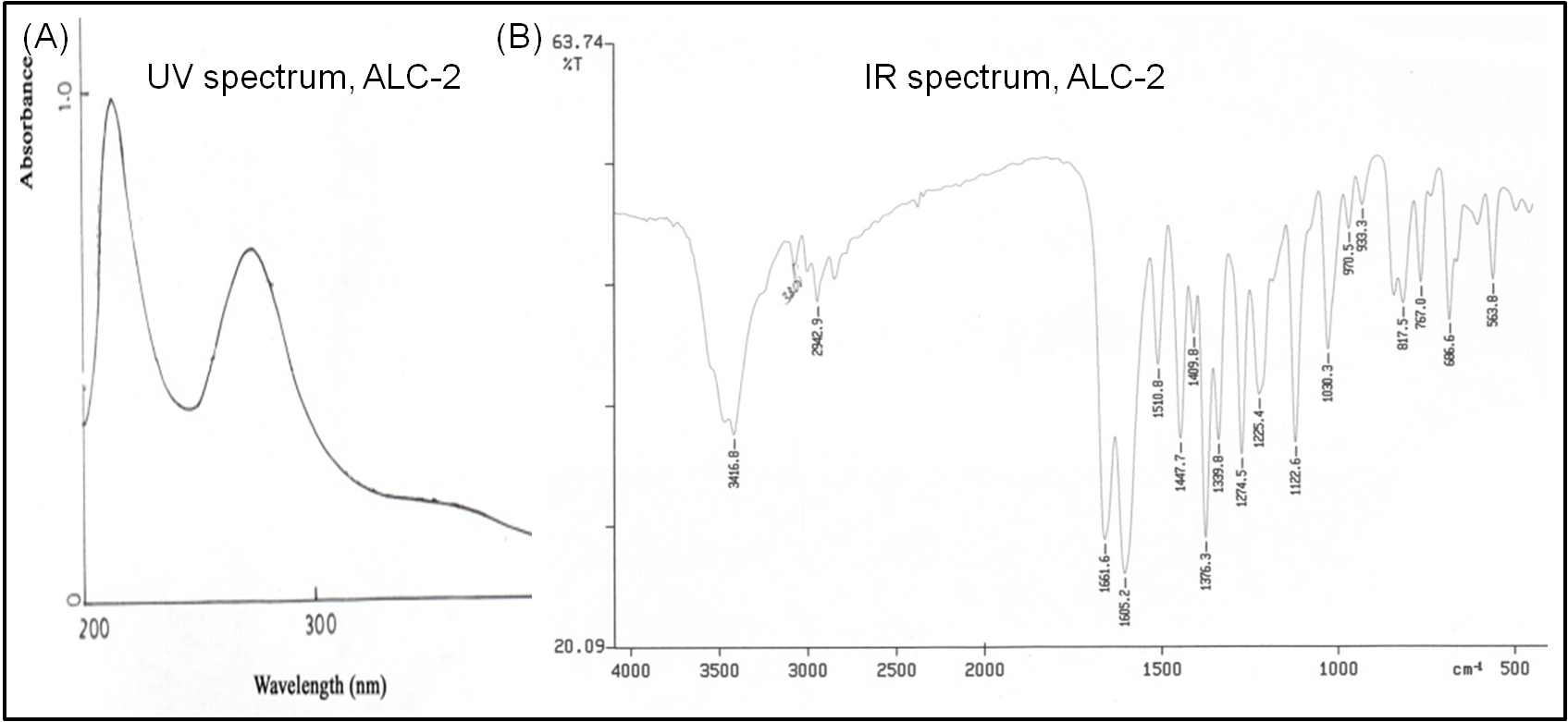

Supplement: S2 Fig — (A) UV spectra (B) IR spectra. (TIF) [file pone.0141154.s002.tif]
